# Styrene maleic-acid lipid particles (SMALPs) into detergent or amphipols: An exchange protocol for membrane protein characterisation

**DOI:** 10.1016/j.bbamem.2020.183192

**Published:** 2020-05-01

**Authors:** Sophie J. Hesketh, David P. Klebl, Anna J. Higgins, Maren Thomsen, Isabelle B. Pickles, Frank Sobott, Asipu Sivaprasadarao, Vincent L.G. Postis, Stephen P. Muench

**Affiliations:** a School of Biomedical Sciences, Faculty of Biological Sciences, University of Leeds, LS2 9JT, UK; b Astbury Centre for Structural Molecular Biology, University of Leeds, LS2 9JT, UK; c Department of Discovery and Translational Science, Leeds Institute of Cardiovascular and Metabolic Medicine, University of Leeds, Leeds LS2 9JT, UK; d School of Molecular and Cellular Biology, Faculty of Biological Sciences, University of Leeds, LS2 9JT, UK; e Department of Chemistry, Biomolecular & Analytical Mass Spectrometry group, University of Antwerp, 2020 Antwerpen, Belgium; f Biomedicine Research Group, Faculty of Health and Social Sciences, Leeds Beckett University, LS1 3HE, UK

**Keywords:** Membrane proteins, SMA *co*-polymer, SMALP, Electron microscopy, Mass spectrometry, Amphipols

## Abstract

Membrane proteins are traditionally extracted and purified in detergent for biochemical and structural characterisation. This process is often costly and laborious, and the stripping away of potentially stabilising lipids from the membrane protein of interest can have detrimental effects on protein integrity. Recently, styrene-maleic acid (SMA) *co*-polymers have offered a solution to this problem by extracting membrane proteins directly from their native membrane, while retaining their naturally associated lipids in the form of stable SMA lipid particles (SMALPs). However, the inherent nature and heterogeneity of the polymer renders their use challenging for some downstream applications – particularly mass spectrometry (MS). While advances in cryo-electron microscopy (cryo-EM) have enhanced our understanding of membrane protein:lipid interactions in both SMALPs and detergent, the resolution obtained with this technique is often insufficient to accurately identify closely associated lipids within the transmembrane annulus. Native-MS has the power to fill this knowledge gap, but the SMA polymer itself remains largely incompatible with this technique. To increase sample homogeneity and allow characterisation of membrane protein:lipid complexes by native-MS, we have developed a novel SMA-exchange method; whereby the membrane protein of interest is first solubilised and purified in SMA, then transferred into amphipols or detergents. This allows the membrane protein and endogenously associated lipids extracted by SMA *co*-polymer to be identified and examined by MS, thereby complementing results obtained by cryo-EM and creating a better understanding of how the lipid bilayer directly affects membrane protein structure and function.

## Highlights

• First reported exchange protocol for transferring membrane proteins solubilised in SMALPs, into detergent or amphipols.
• Purification of protein:lipid complexes without detergent for mass spectrometry and subsequent lipid identification.
• Cost effective membrane protein purification requiring only minimal amounts of detergents in the exchange process.

## 1 Introduction

Despite their physiological importance, our structural, biochemical and biophysical understanding of membrane proteins *in vivo* is limited. This is largely due to the challenges associated with their extraction and subsequent instability outside of their native lipid environment. Traditional *in vitro* membrane protein characterisation methods dictate that the protein is extracted in detergent, which strips away the native membrane and encapsulates the hydrophobic transmembrane region within a micelle to keep it suspended in solution - often delipidating the membrane protein complex in the process. The detergent then remains present throughout all stages of the purification, but can be later exchanged for a different detergent or a more suitable detergent system/solubilisation platform for downstream experimentation. However, optimisation of solubilisation and purification conditions is not trivial, and many resources are frequently committed to this task with limited success. A number of alternative reconstitution platforms have been developed to combat these detergent-associated issues, such as membrane scaffold protein nanodiscs [1,2], amphipols [3,4], peptidiscs [5], bicelles [6] and liposomes [7]; but all still require an initial detergent solubilisation step, often resulting in reduced membrane protein activity and/or detrimental structural perturbations [[8], [9], [10], [11], [12]].

In an attempt to overcome these issues entirely, styrene-maleic acid (SMA) lipid particles (SMALPs) were developed as a platform for membrane protein solubilisation [[13], [14], [15], [16]]. SMA *co*-polymers act by solubilising membrane proteins directly from the membrane while retaining their native lipid environment [13,[15], [16], [17]]. The SMALP:membrane protein complexes then remain intact throughout the purification process and later during storage [11]. Moreover, the SMA *co*-polymer can be easily synthesised in-house by hydrolysis of its precursor anhydride, SMA2000 [16]. This offers a significant advantage over conventional detergents, as it enables solubilisation of a more challenging range of the membrane proteins - such as large complexes with highly integrated lipid structures - at a significantly reduced cost. However, SMALPs are sensitive to low pH (<6.5), divalent cations (> 5 mM) and form heterogeneous complexes [16,18,19]. These properties limit their application in a number of downstream experiments, for example, those which have particular constraints in buffer composition, functional assays and structural techniques including mass spectrometry (MS) and electron microscopy (EM) [20,21]. Derivatives of SMA and alternative *co*-polymers have been designed to overcome issues with sample heterogeneity [22], and susceptibility to pH and divalent cations [19,23,24], but as of yet no one-size-fits-all solution has been established.

We thus propose a more combinatorial approach to *in vitro* membrane protein characterisation, whereby the membrane protein of interest is first extracted in SMALPs, and subsequently exchanged into a more appropriate system for downstream applications. In this context, SMALPs act as a powerful tool for initial solubilisation and purification of membrane proteins, offering a significantly cheaper, stable alternative to conventional detergent purification methods, while retaining lipids from the membrane proteins' native environment.

## 2 Materials and methods

### 2.1 AcrB expression, solubilisation and purification

The styrene-maleic acid (SMA) *co*-polymer (SMA2000, Cray Valley – now trading under Polyscience, N.L) was hydrolysed in-house and stored at 4 °C as a powder stock, as described previously [16]. *Escherichia coli* (*E. coli*) AcrB(His)_8_ was expressed and purified with 2.5% (w/v) SMA as described previously [25], albeit with a few modifications. Briefly, the C43(DE3), pRARE2, ΔacrB strain of *E. coli* was used for overexpression by auto-induction in SB media [26]. Cell membranes were prepared as described in [27], and the membrane pellet was resuspended in a minimal volume of binding buffer (BB: 500 mM NaCl, 10% glycerol, 50 mM Tris-HCl, pH 8.0) before snap-freezing in liquid nitrogen. The Pierce™ BCA protein assay kit (ThermoScientific, U.K.), was used to estimate protein concentration in the isolated membranes. For solubilisation in SMA, membranes were weighed to give ~45 mg total protein and resuspended in BB to an equivalent concentration of 1 mg/ml of protein. SMA *co*-polymer was added to a final concentration of 2.5% (w/v), and the mixture was incubated for 2 h at room temperature with inversion. AcrB purified in n-dodecyl-β-D-maltoside (DDM:AcrB) was solubilised in BB plus 1% DDM and incubated at 4 °C for 2 h. Insoluble material was pelleted by centrifugation at 100,000 xg_av_, 4 °C, and the soluble fractions were incubated with pre-equilibrated HisPur™ cobalt resin (ThermoScientific™, U.K.) overnight at 4 °C. To purify the protein, the resin was first washed with 10 column volumes (CV) of BB (plus 0.025% DDM for DDM:AcrB; DBB), then 10 CV of BB (or DBB) supplemented with 20 mM imidazole, before eluting in 1 ml fractions with elution buffer (BB or DBB plus 300 mM imidazole). Fractions containing the eluted protein (identified by SDS-PAGE) were pooled and dialysed into BB or DBB and then concentrated using an Amicon Ultra 100 kDa MWCO centrifugal filter (Merck Millipore, U.K.), before being snap-frozen in small volumes and stored at −80 °C.

### 2.2 SMA-exchange procedure

For amphipol exchange, A8–35 (5% w/v in H_2_O) was added to SMA-purified AcrB (~0.5 mg/ml for routine exchange, and up to 4.5 mg/ml for SEC-MALLS analysis), at 3:1 w/w amphipol:protein ratio and incubated on ice for 30 min. For detergent exchange, a final concentration of 1% n-dodecyl-β-D-maltoside (DDM) was added to the purified protein sample instead. All sample volumes were ~200 μl or less. After this incubation, the exchange samples and A8–35/DDM-free controls were treated with incremental concentrations (0.5, 1.0, 1.5 and 2.0 mM) of MgCl_2_ to precipitate SMA. Thus, MgCl_2_ was first added to give a final concentration of 0.5 mM, incubated at 4 °C for 1 h with gentle inversion, and then centrifuged at 100,000 xg_av_, 4 °C, for 1 h. The supernatants were transferred to fresh microfuge tubes, and 2 μl aliquots were taken for detection of AcrB(His)_8_ by dot blot. Incubation with MgCl_2_ and subsequent centrifugation steps were repeated four times in total, until the final MgCl_2_ concentration reached 2 mM. The A8–35 MgCl_2_-free controls were also subject to the same periods of incubation and centrifugation for consistency. The final product was taken for further analysis by SEC-MALLS, negative stain electron microscopy, and mass spectrometry. Samples were exchanged into the appropriate buffers for each application before use.

### 2.3 Dot blot

Supernatant samples were mixed 1:1 (v/v) with 0.1% SDS and 25 mM DTT and incubated at room temperature for a minimum of 5 min. Samples were spotted directly onto nitrocellulose membranes (0.45 μM pore size) in a 2 μL volume and left to air-dry. Blots were blocked with 5% Marvel milk in PBST (13. 7 mM NaCl, 0.27 mM KCl, 1 mM Na_2_HPO_4_.7H_2_O, 0.18 mM KH_2_PO_4_ and 0.05% Tween 20), for 1 h at room temperature. Membranes were rinsed twice in PBST for 5 min, before submerging in 2% Marvel milk in PBST supplemented with 1:4000 diluted HRP-conjugated monoclonal mouse anti-His antibody (R&D Systems, U.K.) for a further 1 h at room temperature. Blots were then washed for 1 h with PBST before developing.

### 2.4 Size exclusion chromatography with multi-angle laser light scattering (SEC-MALLS)

SMA:AcrB and A8–35 exchanged (A8–35_Ex) samples were dialysed into 300 mM NaCl, 50 mM Tris-HCl, pH 8.0 (SECB) prior to SEC-MALLS experimentation. DDM exchanged (DDM_Ex) samples were dialysed into SECB plus 10% glycerol and 0.025% DDM (DDM-SECB) to decrease the DDM concentration. For the protein-free lipid particles, 5 mg of *E. coli* total lipid extracts (Avanti Polar Lipids Inc., U.S.A.) were suspended by sonication in SECB, and split into two for the addition of 2.5% SMA and 2.5% A8–35. SEC-MALLS experiments were performed using a Superose 6 5/150 column pre-equilibrated with SECB for SMA:AcrB and A8–35_Ex samples, and DDM-SECB without glycerol for DDM-containing samples. The data were collected on a DAWN 8+ multi-angle light scattering (LS) detector, an Optilab T-rEX differential refractive index (dRI) detector and UV-absorbance (UV) detector (Wyatt Technology), and samples were run at a flow rate of 0.2 ml/min. Astra 6.2 software was implemented for molar mass calculations. The absorbance value at 0.1% OD_280nm_ for full-length AcrB(His)_8_ was given as 0.79 g/l and a refractive index increment (dn/dc) value of 0.185 ml/g was applied to the protein component - AcrB. A8–35 and DDM dn/dc modifiers were set at 0.15 and 0.143, respectively, for the surfactant components.

### 2.5 Negative stain electron microscopy

Negative stain grids were prepared and examined as previously described [20]. Briefly, in-house carbon-coated copper grids were glow-discharged (PELCO easiGlow, TedPella) for 30 s. 3 μl of sample at a concentration of ~20 μg/ml was then applied to the grid for 30 s and blotted before staining twice with 1% uranyl acetate (2× 30s). Micrographs were collected using a Tecnai F20 microscope fitted with a 4 k × 4 K CMOS camera, operating at 200 kV with a nominal magnification of 50,000×.

### 2.6 Lipid extraction and denaturing mass spectrometry

Lipid extraction was performed as described in [28], albeit with the following modifications. All steps of lipid extraction were performed on ice or at 4 °C. To 40 μl A8–35_Ex (1 volume; [AcrB] ~1.5 mg/ml), 1 volume of chloroform and 2 volumes of methanol were added. The sample was mixed and another volume of chloroform was added. After mixing again, 1 volume of water was added. The sample was centrifuged (7 min, 17,000 xg), before the organic phase was washed three times with 2 volumes of cold water. The organic phase was directly analysed by nano-electrospray ionisation (nESI)-MS using in-house coated gold/palladium nanospray capillaries and a quadrupole time-of-flight MS (Synapt G1 HDMS, Waters) operating in negative mode. For denaturing MS of lipid extracts, the synapt was operated with the following parameters: Capillary voltage = 1.2 kV, source temperature = 80 °C; sampling cone = 80 V; extraction cone = 4 V; backing pressure = 2 mbar; trap collision energy (CE) = 20 V; trap flow rate = 2 ml/min, transfer CE = 10 V and trap DC bias = 4. The most intense signal (719 *m*/*z*) was selected and fragmented by MS/MS under the same conditions, except with trap CE = 50 V.

### 2.7 Native mass spectrometry

Samples were prepared for MS by diluting with 200 mM ammonium acetate, pH 7.4, and re-concentrating with an Amicon Ultra 0.5 ml (100 kDa MWCO) concentrator. This was repeated at least three times to ensure buffer exchange. Native MS was done using nESI with in-house prepared nanospray capillaries on a Synapt G1, operated in positive ion mode. The instrument parameters were as follows: Capillary voltage = 2.0 kV; source temperature = 80 °C; sampling cone = 180 V; extraction cone = 4 V; backing pressure = 6 mbar; trap CE = 220 V; transfer CE = 200 V and trap DC bias = 4. All MS data were analysed with MassLynx software (Waters).

## 3 Results and discussion

### 3.1 SMA-exchange procedure

The SMA-exchange procedure was designed to capitalise on the polymers' inherent sensitivity to divalent cations, whereby MgCl_2_ is used to gradually destabilise the SMA polymer and thus promote protein incorporation into an alternative platform – such as detergent or another amphipathic polymer. The *E. coli* multidrug efflux pump, acridine resistance protein B (AcrB), was chosen as a model system to test the exchange, as it has been previously characterised by a variety of biochemical and biophysical techniques in detergents [29,30], amphipols [31] and SMALPs [25,32].

**Fig. 1:**
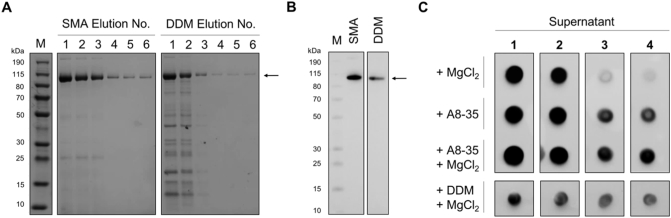
Solubilisation and purification efficiency of SMA *vs* DDM for AcrB (A and B), alongside a dot blot monitoring the exchange procedure from SMA into amphipol A8–35 and DDM (C). A) Representative elution fractions (1–6) from a standard one-step IMAC purification of AcrB in SMA and DDM alongside molecular weight marker (M). Arrow indicates the expected molecular mass of AcrB. B) Anti-His western blot of final AcrB(His)_8_ samples in SMA and DDM with overlaid molecular weight marker (M) prior to further experimentation. C) Dot blot showing the relative amount of soluble AcrB remaining in the supernatant after each incubation with the precipitant (MgCl_2_), rescue agent (A8–35 or DDM), or a combination of both. Supernatant 1 represents the sample after incubation and centrifugation with the first MgCl_2_ addition, in this case 0.5 mM MgCl_2_. Supernatants 2–4 represent the remaining soluble AcrB after each 0.5 mM increment of MgCl_2_ added to the sample. The same antibody was used as in B.

AcrB was extracted and purified in SMALPs (SMA:AcrB) using a one-step IMAC cobalt purification, as previously described [25]. Purification was also performed in n-dodecyl-β-D-maltoside (DDM) for comparison (DDM:AcrB) (Fig. 1A and B). AcrB purified using the SMA *co*-polymer routinely yielded a purer sample in the elution fractions, thus limiting the number of downstream steps required to purify the sample further. This prevents unnecessary protein loss as a result of additional purification steps, which in turn increases cost-effectiveness.

To destabilise SMA:AcrB and encourage exchange, the purified sample was first dialysed into BB, then incubated with (0.5 mM) MgCl_2_ for 1 h in the presence or absence of either amphipol (A8–35) or DDM. Although it has been shown that amphipols are also sensitive to divalent cations, they are relatively more tolerant to Mg^2+^ than the SMA *co*-polymer, which starts to heavily precipitate at 2 mM MgCl_2_ in this instance (Fig. 1C) [19,33]. It is likely that using an alternative amphipathic polymer or detergent (e.g. DDM) which is less sensitive to divalent cations would perform better in the exchange than the A8–35 polymer tested here. Alternatively, a polymer variant that is more sensitive to divalent cations, such as SMA3000, may increase the efficiency of the exchange process [34]. After the first incubation with 0.5 mM MgCl_2_, insoluble material was pelleted by centrifugation at 100,000 x*g*, before increasing the MgCl_2_ concentration by a further 0.5 mM and incubating for another hour. These steps were repeated until the mixture reached a final concentration of 2 mM MgCl_2_. Dot blots were used to monitor the quantity of soluble AcrB remaining in the supernatant throughout the exchange process with the aim of keeping sample use to a minimum (Fig. 1C), but for clarity the A8–35 exchange samples were also analysed by standard western blotting (Supplementary Fig. 1E).

At 2 mM MgCl_2_, AcrB remains soluble in the presence of A8–35 or DDM, but completely precipitates in the absence of such a rescue agent (Fig. 1C). A MgCl_2_-free control was also included to examine the effect of the competing A8–35 polymer alone (Fig. 1C, +A8–35). The final protein concentration in the +A8–35 samples are similar to that of the exchanged sample (Supplementary Table 1), suggesting that the presence of a rescue agent alone does not destabilise the SMA:AcrB complex and cause precipitation. A small sample loss can be observed during both amphipol and detergent exchange experiments, which may be attributed to the instability caused by the presence of MgCl_2_. However, as the goal of the exchange is to remove the SMA copolymer, the presence of MgCl_2_ as a precipitant is necessary to assist in exchange efficiency. It is also noteworthy that the nature of the transmembrane annulus after addition of the exchange material in the presence or absence of MgCl_2_ – whether a complete exchange has occurred, or a polymer/detergent hybrid has formed – is unknown. In future, although beyond the scope of this study, the use of fluorescently labelled SMA and amphipols could be used to quantitatively determine the exchange efficiency and give more insight into the true amount of remaining SMA polymer [35,36]. Additionally, while the MgCl_2_ concentration used here has been shown to precipitate the polymer and the associated protein out of solution (Fig. 1C, +MgCl_2_), the required concentration for precipitation is likely to be protein dependent and should be empirically determined for different systems. However, it has been shown that the sensitivity to divalent cations is likely polymer-specific [37]. It is also noteworthy that the volume of the reaction and concentration of the protein may play a role in exchange efficiency, and these experiments were designed with low protein concentrations in ~200 μl volumes in mind.

### 3.2 Validation of exchange and examining polymer influence with SEC-MALLS

SMA *co*-polymers have an average polydispersity index of ~2.6 [38,39]. This polymer heterogeneity makes membrane protein:SMA particle characterisation difficult, particularly when used in conjunction with techniques that prioritise sample homogeneity [23]. There have been attempts to decrease the heterogeneity of the polymer by altering the synthesis procedure and polymer length, but it has been suggested that the SMA *co*-polymer's solubilisation efficiency is owed to this heterogeneity [40]. This protocol was therefore designed to reduce sample heterogeneity without interfering with the original polymer synthesis procedure.

**Fig. 2:**
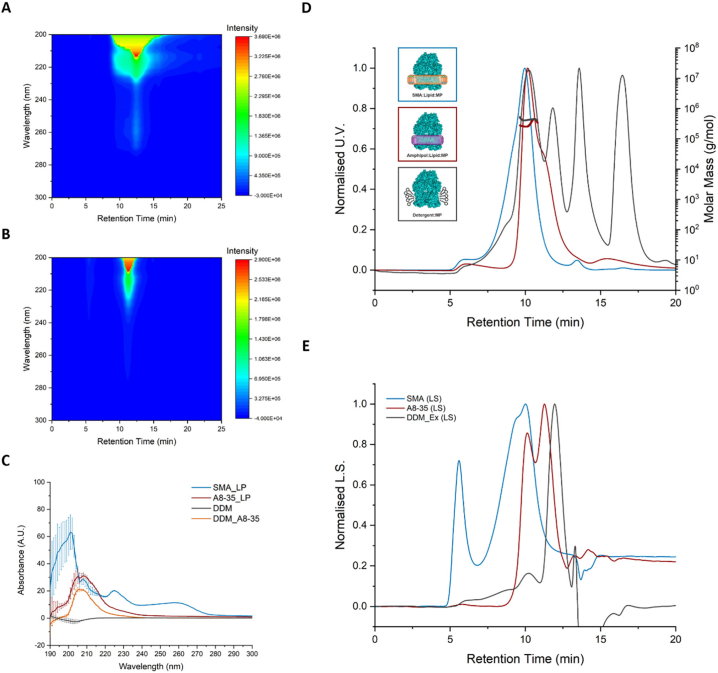
SEC-MALLS analysis of exchanged samples and evidence of polymer interference across a range of UV wavelengths. A) Multi-wavelength SEC profile of protein-free SMA lipid particle (SMA_LP) in heat map representation. Relative absorbance at each wavelength is shown as intensity, ranging from highest absorbance (red) to the lowest (blue), and plotted according to retention time in minutes. B) As A, but with amphipol A8–35 lipid particles (A8–35_LP). C) UV absorbance of DDM alongside SMA_LP and A8–35_LP from A and B. A8–35 was also mixed with DDM (DDM_A8–35) to give clarification on the polymer and lipid contributions to absorbance across a range of wavelengths. Error bars represent triplicate results. D) SEC-MALLS chromatograms and molar mass distributions of SMA:AcrB (blue), A8–35_Ex (red) and DDM_Ex (grey). Images representing the different solubilisation platforms from Fig. 1 have been colour-coded according to their related SEC profiles. E) Light scattering (LS) profiles of the same samples shown in D, colour-coded according to D. Traces in D) and E) were normalized relative to the highest peak (Normalized U.V./L.S.) and plotted as a function of retention time in minutes (min).

To examine the effect that SMA and A8–35 can have on the polydispersity of a sample, protein-free SMA lipid particles (SMA_LP) and A8–35 lipid particles (A8–35_LP) were examined by size exclusion chromatography with multi-angle laser light scattering (SEC-MALLS), and their absorbance was monitored across multiple wavelengths (Fig. 2A and B). SMA_LP significantly absorbed in the UV spectrum from ~200–270 nm, with a gradual drop in absorbance before 280 nm. The SMA_LP also eluted slowly from the SEC column, visualised as a broad primary elution peak spanning >5 min of the total 20-min run. The A8–35_LP did not display similar characteristics, and instead eluted in a sharper peak spanning ~2.5 min of retention time. This highlights a possible advantage of using amphipols as a homogeneous tool instead of SMA for techniques such as MS. The absorbance spectra of SMA_LPs, A8–35_LPs were measured alongside DDM and a DDM-A8–35 mix in triplicate to validate these absorbance readings (Fig. 2C).

**Fig. 3:**
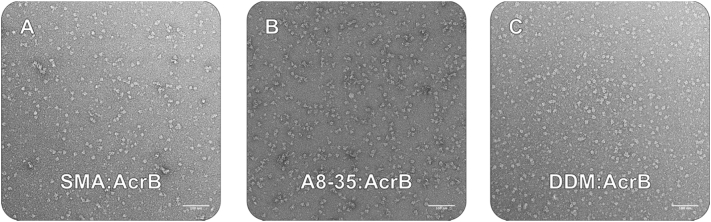
Negative stain analysis of AcrB after extraction in SMA and its subsequent exchange in A8–35 and DDM. Representative negative stain micrographs of SMA:AcrB before (A) and after exchange into A8–35 (B) and DDM (C). In each instance the “typical” broadly triangular shape can be seen with no significant aggregation. Micrographs were taken at 50 k mag. Bottom-right scale bar = 100 nm.

Exchanged AcrB samples were also analysed by SEC-MALLS to determine whether sample homogeneity had improved. Comparison of the SEC-MALLS chromatograms of SMA:AcrB and the A8–35 exchanged (A8–35_Ex) sample showed a reduction in heterogeneity between the starting material and final exchanged product, indicated by a leading trail before the primary elution peak for SMA:AcrB, and a sharp elution peak for A8–35_Ex at ~10 min (Fig. 2D and E); and is further demonstrated by a more consistent molar mass distribution of A8–35_Ex across this peak (Fig. 2D, Supplementary Fig. 2A). Aggregates of high laser light scattering (LLS; Fig. 2E) and low UV absorbance were also observed in the void volume for the SMA:AcrB sample, potentially indicating the presence of polymer-only aggregates. No such aggregation was observed in the void volume for A8–35_Ex, but a large LLS peak corresponding to the size of empty A8–35 lipid particles was consistently observed after the exchange at an elution time of ~11.35 min [41] (Figs. 2B and 3E). Accurate molar masses could not be determined for SMA:AcrB due to discrepancy in the literature describing the refractive index increment (dn/dc) for SMA *co*-polymers [15,22,34], but a dn/dc polymer modifier of 0.15 [41] could be applied to the A8–35 sample to give an estimated molar mass of ~450 kDa at the highest peak (Fig. 2A and Supplementary Fig. 2A). Analytical ultracentrifugation has previously determined the molecular mass of AcrB in SMA to be >400 kDa [42], although it has been observed at variable molecular weights ranging from its native ~340 kDa up to and exceeding 800 kDa [42,43].

AcrB was also exchanged into DDM (DDM_Ex) and analysed alongside a control sample that had been solubilised and purified in DDM by conventional methods (Fig. 1A, Supplementary Fig. 1B). No significant aggregation was observed in the SEC-MALLS elution profile of either sample. The DDM_Ex elution peak also has a slight decrease in retention time (~ 10.2 min) compared to the DDM purified sample (~10.4 min, supplementary information). Despite this peak shift, the primary elution peak for DDM_Ex at 10 min is still less broad. However, a modifier dn/dc value of 0.143, corresponding to the DDM micelle RI, was applied to both samples, and gave a similar molar mass distribution (~400 kDa) to the A8–35_Ex sample. The molar mass distribution shows a homogeneous mixture at this point and the reason for this is unclear but could again be due to the unclear nature of the lipid annulus.

### 3.3 Validation of exchange: negative stain microscopy

Negative stain microscopy was used to visually assess the homogeneity and integrity of the exchanged samples *vs* the progenitor SMA:AcrB sample (Fig. 3). Both DDM_Ex and A8–35_Ex were monodisperse and homogeneous on the grid, and particles clearly maintained the characteristic AcrB architecture, which is typically seen as a broadly triangular shape [29,32,44]. Although negative stain can report on the overall quality of the protein sample in terms of aggregation and degradation, the resolution is not sufficient to see subtle changes in structure or discern the nature and lipid content of the annulus surrounding the protein of interest.

### 3.4 Validation of exchange: mass spectrometry

One significant advantage of adopting a SMALP method is that it can extract and isolate the protein of interest in the presence of its native lipid annulus [13,[15], [16], [17]]. Recently, MS has emerged as a powerful complementary tool for examining the extracted membrane protein:lipid complexes, both in native-MS to determine lipid stoichiometry [12,45,46], and LC-MS/MS to examine their lipid profile [43,47]. However, the heterogeneity of the SMA polymer and its inability to dissociate easily in the gas phase has proved problematic for native-MS studies of SMA:membrane protein complexes, as the SMA *co*-polymer produces a large variety of charge states with complex drift time measurements. Exchanging the bound SMA with a detergent or amphipol may overcome this issue, as both amphipols [48,49] and detergents [45,50] have been extensively characterised in MS [51,52]. It has also been previously shown that A8–35:membrane protein complexes out-perform their detergent counterparts, so it would be beneficial for the membrane protein to have been fully exchanged into this system [53]. Additionally, subsequent lipidomic assessment of A8–35_Ex and DDM_Ex samples would complement native-MS results. To this end, we compared A8–35_Ex and DDM_Ex in native MS to first examine the complexes individually, and then used tandem mass spectrometry (MS/MS) to identify the presence of any remaining lipids.

**Fig. 4:**
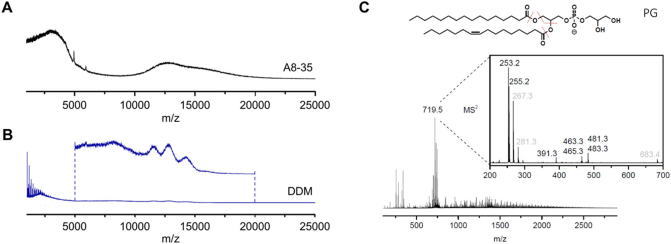
Mass spectrometry analysis of SMA extracted AcrB after exchange into A8–35 and DDM. Initial native MS results of A8–35_Ex (A) and DDM_Ex (B). C) Tandem mass spectra (MS/MS) of lipids extracted from A8–35_Ex, demonstrating that at least one lipid can be identified as phosphatidylglycerol (PG). The preferential fragmentation positions of PG (16:0/16:1) are indicated by red lines. MS/MS-peaks which could not be explained were labelled in grey.

MS analysis showed that the exchange of the protein from SMA to both A8–35 and DDM was achieved to some degree, as firstly, spectra had been observed where this was not previously obtainable for SMA:AcrB; secondly, the observed spectra are similar to that of the published detergent:AcrB spectrum [50] (Fig. 4A and B). These traces were of low resolution, even at high collision energies, which may suggest that a small amount of SMA *co*-polymer remains bound but overall demonstrate that a more homogeneous, tractable sample had been generated.

Based on previous evidence of lipid retention in SMA [23,47], we also hypothesised that native lipids were carried through during the transfer from SMA:AcrB to A8–35_Ex, as the protein was not initially extracted in delipidating conditions. DDM is known to delipidate membrane proteins for mass spectrometry, but this information is not available for amphipols [45]. After the exchange, lipids of the non-detergent exposed A8–35_Ex sample were extracted and the crude extract directly subjected to MS analysis. The resulting spectra show a large number of putative lipid peaks. The most intense peak at *m*/*z* = 719.5 was taken forward to tandem MS and assigned to phosphatidylglycerol (16:0/16:1) according to previously published data [54]. This was further validated using LIPID MAPS glycerophospholipid MS/MS prediction [55] (Fig. 4C). Albeit a very preliminary result, this shows that lipids can be retained through this entirely detergent free purification and exchange procedure and provides a base for future investigation into how the relationship between solubilising agent (polymer/detergent) can define the nature of the lipid annulus, and thus how membrane proteins interact with their native bilayer.

## 4 Conclusions

**Fig. 5:**
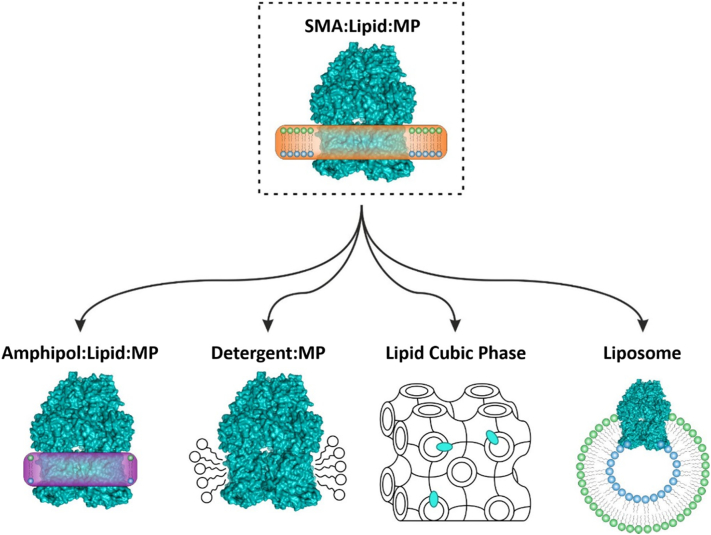
SMALPs as a vehicle for transfer into downstream membrane protein solubilisation platforms. Schematic highlighting the versatility of the SMA *co*-polymer as a vehicle for reconstitution into downstream solubilisation platforms amphipol, detergents, lipid cubic phase and liposomes.

Here, we present a method of platform exchange for membrane proteins, purified with the SMA *co*-polymer, to enhance compatibility with downstream applications, such as MS, EM and SEC. The method capitalises on the ability of MgCl_2_ to precipitate out the SMA co-polymer in the presence of an alternative solubilisation platform, such as detergents or amphipols. A further advantage is that it allows native lipids to be carried through the initial protein solubilisation/purification steps, which enhances the stability of the membrane protein in solution. Additionally, as detergents and amphipols are not required in the membrane protein preparation until the final exchange step, it can significantly reduce the costs commonly associated with standard detergent purifications. AcrB was used as a model system to test the exchange process, as it is extensively characterised in a number of reconstitution platforms. To this end, we successfully purified AcrB with the SMA co-polymer and exchanged the polymer for an alternative platform – namely, amphipol, A8–35 and detergent, DDM. The final exchange product was more homogeneous, could withstand millimolar concentrations of MgCl_2_ and gave observable spectra in native-MS analysis, which were our initial objectives. The exchange process can also be performed on a small scale with minimal sample loss, which is beneficial to more difficult systems where low protein concentrations are obtained. We also were able to use mass spectrometry to present evidence that lipids are carried over from the transfer of SMA into A8–35, which opens up the possibility of analysing native membrane protein:lipid complexes extracted and purified in the complete absence of detergent alongside their complimentary lipid profiles. Overall this method has the potential to provide further insights into native membrane protein:lipid interactions by adding to the ever-expanding SMA toolbox for membrane protein characterisation (Fig. 5).

## Declaration of competing interest

The authors declare that they have no known competing financial interests or personal relationships that could have appeared to influence the work reported in this paper.

## Appendix A Supplementary data

Supplementary material Image 1
