# Supplementary material for: Styrene maleic-acid lipid particles (SMALPs) into detergent or amphipols: An exchange protocol for membrane protein characterisation
Source: Biochim Biophys Acta Biomembr. 2020 May 1;1862(5):183192. doi: 10.1016/j.bbamem.2020.183192 (PMC7086155; doi:10.1016/j.bbamem.2020.183192)
Supplement: Supplementary file 1 — Supplementary material [file mmc1.pdf]

Supplementary Information

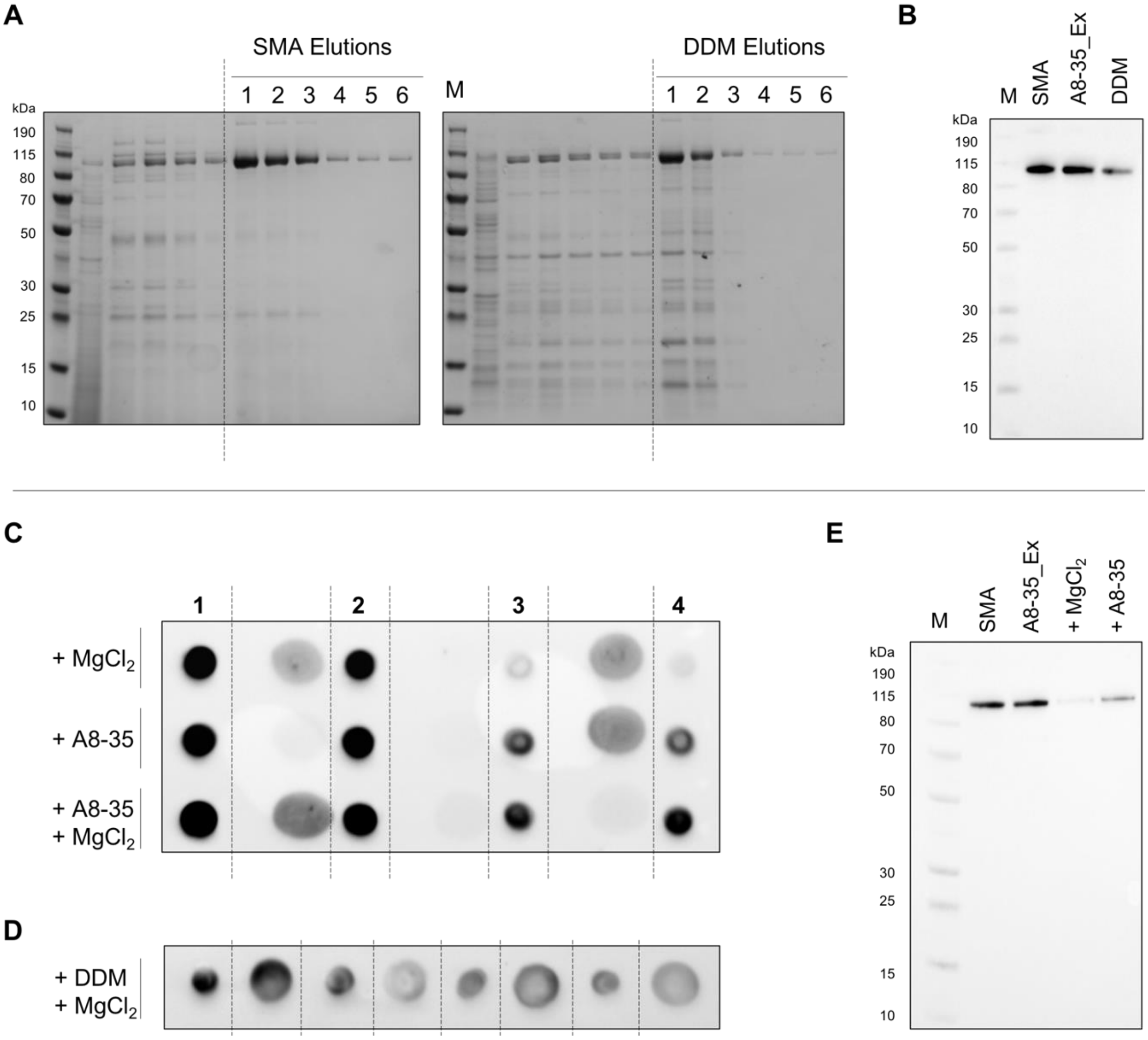

Supplementary Figure 1: A) Full gel images of SMA and DDM purifications of AcrB with accompanying molecular weight markers (MWM). **B)** Full western blot image of SMA and DDM purified AcrB samples with A8-35_Ex sample in middle lane (omitted in final image in Figure 1B), alongside the same MWM as in A. **C)** Complete dot blot of amphipol A8-35_Ex samples – dotted lines represent boxed regions shown in Figure 1C. The larger regions in between represent other samples. **D)** Full dot blot of DDM_Ex samples – dotted lines represent boxed regions shown in Figure 1C. **E)** Western blot of complete exchange process showing original SMA sample (SMA), the final exchanged sample (A8-35_Ex), the $MgCl_2$-only control (+$MgCl_2$), and A8-35-only control (+A8-35), alongside MWM as in A.

A

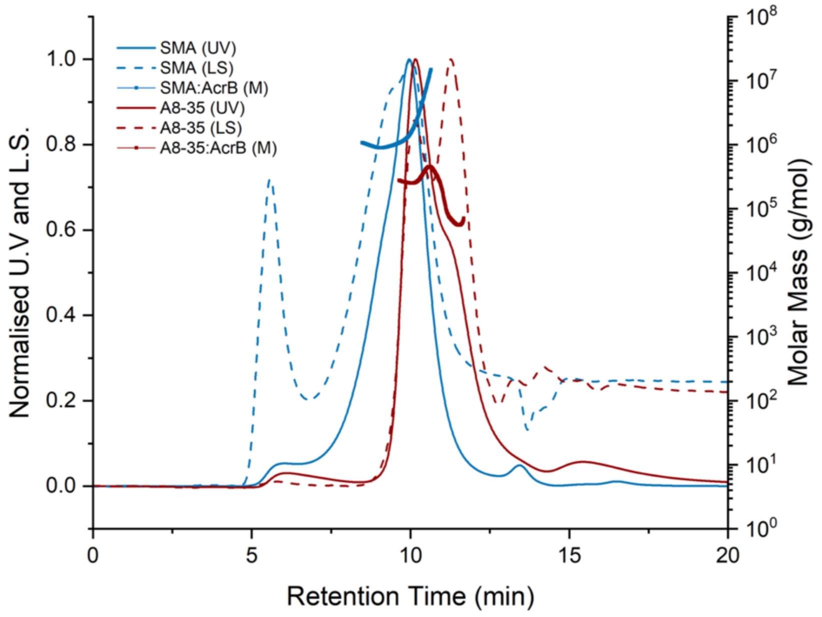

B

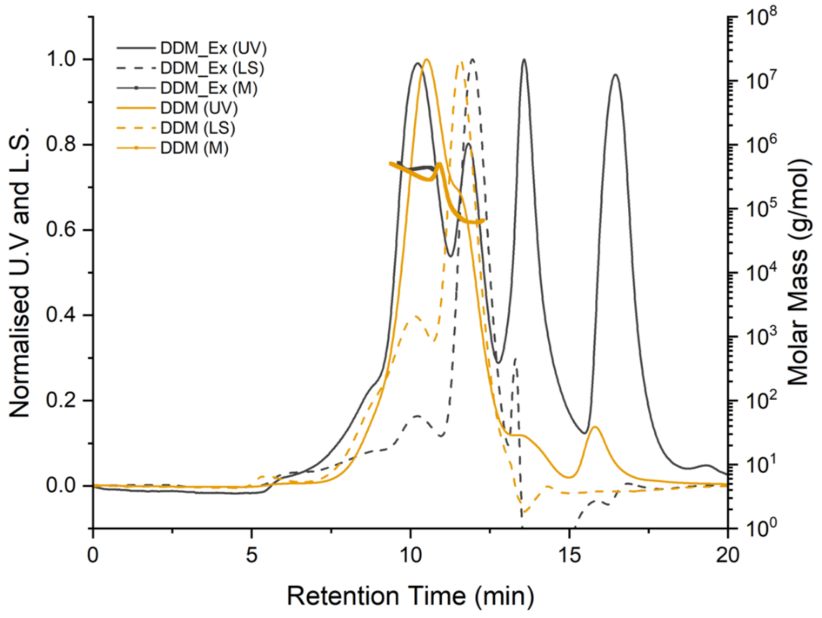

Supplementary Figure 2: A) SEC-MALLS analysis of SMA:AcrB (blue) and A8-35_Ex (red). Solid lines represent UV absorbance at 280 nm and dashed lines represent light scattering signal. Traces were normalized relative to the highest peak (Normalized U.V.), and plotted as a function of retention time in minutes. A refractive index increment (dn/dc) value of 0.16 mL/g from [1] was applied for SMA to give the molar mass distribution (shown as a line across the peak), but there were issues with the calculations and this data is included for relative comparisons of heterogeneity only. **B)** SEC-MALLS analysis of DDM:AcrB (yellow) and DDM_Ex (grey). Solid lines represent UV absorbance at 280 nm and dashed lines represent light scattering signal. Traces were normalized relative to the highest peak (Normalized U.V.), and plotted as a function of retention time in minutes. Calculated molar mass distributions are shown across the entire peak for both samples and are coloured according to each sample as described in A.

[56] Hall S.C.L., et al., Influence of poly(styrene-com-aleic acid) copolymer structure on the properties and self-assembly of SMALP nanodiscs, Biomacromolecules 19 (2018) 761–772.

Sample Name	$MgCl_2$	A8-35	Protein Before (µg)	A8-35 Added (µg)	Protein Conc. After (mg/ml)	Protein After (µg)	Fold Reduction
Control 1	+	-	72.0	-	0.229	2.9	24.83
Control 2	-	+	116.8	350.4	0.335	41.8	2.79
Sample 1	+	+	116.8	350.4	0.340	42.5	2.75
Sample 2	+	+	116.8	350.4	0.327	40.8	2.86
Sample 3	+	+	196.0	588.0	0.789	73.6	2.66

Supplementary Table 1: Conditions of each sample at the start of the experiment are shown in the leftmost columns in green, where control 1 is AcrB with $MgCl_2$ alone, control 2 is A8-35 alone, and samples 1, 2 and 3 are repeats of the amphipol exchange experiment containing both $MgCl_2$ and A8-35. The final sample conditions measured after the exchange are shown in the rightmost columns in blue.
